# Astrocytic HILPDA promotes lipid droplets generation to drive cognitive dysfunction in mice with sepsis‐associated encephalopathy

**DOI:** 10.1111/cns.14758

**Published:** 2024-05-16

**Authors:** Ling Li, Du Lixia, Guifen Gan, Jin Li, Lin Yang, You Wu, Zongping Fang, Xijing Zhang

**Affiliations:** ^1^ Department of Critical Care Medicine Xijing Hospital Fourth Military Medical University Xi'an Shaanxi China; ^2^ Department of Anesthesiology and Perioperative Medicine and Department of Intensive Care Unit Xijing Hospital Fourth Military Medical University Xi'an Shaanxi China; ^3^ Department of Pediatric Xijing Hospital Fourth Military Medical University Xi'an Shaanxi China; ^4^ Department of Critical Care Medicine Qinghai University Affiliated Hospital Xining Qinghai China; ^5^ Translational Research Institute of Brain and Brain‐Like Intelligence Shanghai Fourth People's Hospital School of Medicine Tongji University Shanghai China; ^6^ Department of Critical Care Medicine Shanghai Fourth People's Hospital School of Medicine Tongji University Shanghai China

**Keywords:** astrocyte, cognitive dysfunction, HILPDA, lipid droplets, sepsis‐associated encephalopathy

## Abstract

**Aims:**

Sepsis‐associated encephalopathy (SAE) is manifested as a spectrum of disturbed cerebral function ranging from mild delirium to coma. However, the pathogenesis of SAE has not been clearly elucidated. Astrocytes play important roles in maintaining the function and metabolism of the brain. Most recently, it has been demonstrated that disorders of lipid metabolism, especially lipid droplets (LDs) dyshomeostasis, are involved in a variety of neurodegenerative diseases. The aim of this study was to investigate whether LDs are involved in the underlying mechanism of SAE.

**Methods:**

The open field test, Y‐maze test, and contextual fear conditioning test (CFCT) were used to test cognitive function in SAE mice. Lipidomics was utilized to investigate alterations in hippocampal lipid metabolism in SAE mice. Western blotting and immunofluorescence labeling were applied for the observation of related proteins.

**Results:**

In the current study, we found that SAE mice showed severe cognitive dysfunction, including spatial working and contextual memory. Meanwhile, we demonstrated that lipid metabolism was widely dysregulated in the hippocampus by using lipidomic analysis. Furthermore, western blotting and immunofluorescence confirmed that LDs accumulation in hippocampal astrocytes was involved in the pathological process of cognitive dysfunction in SAE mice. We verified that LDs can be inhibited by specifically suppress hypoxia‐inducible lipid droplet‐associated protein (HILPDA) in astrocytes. Meanwhile, cognitive dysfunction in SAE was ameliorated by reducing A1 astrocyte activation and inhibiting presynaptic membrane transmitter release.

**Conclusion:**

The accumulation of astrocytic lipid droplets plays a crucial role in the pathological process of SAE. HILPDA is an attractive therapeutic target for lipid metabolism regulation and cognitive improvement in septic patients.

## Graphical abstract

Representative schematic of the hypothesis. The accumulation of LDs in hippocampal astrocytes is one of the pathological mechanisms underlying cognitive dysfunction in SAE mice. Increased expression of HILPDA induing astrocyte LDs accumulation contributes to cognitive dysfunction in SAE mice by accelerating the conversion of astrocytes to the A1 phenotype and neuronal membrane potential excitability.

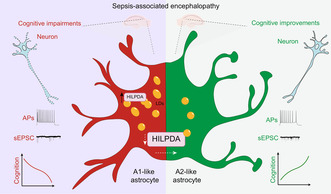

## 1 INTRODUCTION

Sepsis is a life‐threatening syndrome that is caused by dysregulation of the host response to infection and leads to organ failure.^1^ As a common complication of sepsis, sepsis‐associated encephalopathy (SAE) is defined as a syndrome of cerebral dysfunction secondary to sepsis but in the absence of direct infection of central nervous system.^2^ It has been reported that SAE occurs in approximately 70% of patients with sepsis.^3^ Furthermore, clinical studies have shown that more than 60% of survivors suffer from cognitive deficits and memory impairment.^4^ Meanwhile, the hippocampus exhibits pronounced atrophy and significantly reduced volume in survivors of SAE.^5^ However, the underlying mechanism of SAE is still unclear. Blood–brain barrier dysfunction, neuroinflammation, and neurotransmitter abnormalities have been considered to be associated with SAE, but drugs targeting the above mechanisms have not been successfully used in clinical practice.^6^

Lipids are extremely rich in the brain, which plays important roles in maintaining the function of the brain. It has been reported that lipids account for 50%–60% of the dry weight of the brain and are essential for maintaining neuronal homostasis.^7^ Among the cells in the brain, astrocytes are the most dominant cell type^8^ and play important roles in lipid synthesis.^9^ Meanwhile, astrocytes can communicate with neurons via fatty acid metabolism to maintain parenchymal structure and energy metabolism.^10^ Disruption of the normal functions of lipids leads to the development of several neurodegenerative diseases.^11^ However, the role of lipid metabolism in hippocampal astrocytes in the context of SAE is not fully elucidated. Moreover, lipid droplets (LDs) have emerged in recent years as fully entitled organelles with key functions in lipid and energy homeostasis.^12^ There is evidence that inflammation and oxidative stress can lead to an increase in lipid synthesis in neurons and accumulation of lipid droplets in astrocytes.^13^ And the abnormal accumulation of LDs in astrocytes has been proven to be associated with cognitive impairments in Alzheimer's disease model.^14^ Hypoxia‐inducible LD‐associated protein (HILPDA) consists of 63 amino acids and is located on the superficial of LDs.^15^ Previous studies have proved that the up‐regulation of HILPDA caused by hypoxia and stress is highly correlated with the increase of LDs formation and the inhibition of LDs degradation.^16^, ^17^, ^18^ Therefore, we hypothesis that LDs may related to SAE development, and HILPDA can be a intervention or therapeutic target.

In the current study, we found that the homeostasis of lipid metabolism is dramatically altered in hippocampus. Moreover, HILPDA mediates the astrocytic accumulation of LDs. Specifically, downregulating astrocytic HILPDA can attenuate cognitive dysfunction in SAE mice by decreasing the conversion of astrocytes to the A1 phenotype and reversing neuronal membrane potential excitability. Our findings establish a distinct role for astrocytic LDs and HILPDA in mediating the key pathological process of SAE.

## 2 MATERIALS AND METHODS

### 2.1 Animals and study design

All the experiments were designed and performed in accordance with the guidelines of the Fourth Military Medical University (approval #: IACUC‐20210260). Six‐ to eight‐week‐old male wild‐type C57/BL6 mice were purchased from Charles River Co., Ltd. (China, Beijing). The mice were housed in an environment at 22°C–25°C in 55%–58% relative humidity on a 12‐hour light/dark cycle. Food and water were provided ad libitum. In our experiment, all the animals were randomly grouped. In the behavioral paradigm experiment, the mice were divided into the ACSF group and 72 h LPS group. In the lipid metabolism sequencing assay, the mice were divided into the ACSF group and 24 h and 72 h LPS groups. Western blotting analysis was performed to determine LDs levels in the hippocampus at 12 h, 24 h, 48 h, and 72 h after LPS treatment. The knockdown virus experiment included the control AAV‐ACSF group, control AAV‐LPS 72 h group, shHILPDA‐ACSF group, and shHILPDA‐LPS 72 h group.

### 2.2 Antibodies and virus

The following primary antibodies were used in this study: rabbit anti‐plin2 antibody (Abcam, ab108323), rabbit anti‐BCSL2/Seipin antibody (Abcam, ab106793), mouse anti‐HSP90 monoclonal antibody (Proteintech, 60,318–1‐1 g), mouse anti‐HILPDA antibody (Santa Cruz, SC‐376704), goat anti‐Iba1 antibody (Abcam, ab48004), mouse anti‐Neun antibody (Abcam, ab104224), Chicek anti‐GFAP antibody (Genetex, GTX85454), rabbit anti‐S100A10 (Invitrogen, PA5‐95505), and goat anti‐C3d antibody (Bio‐techne, VFL0521021). The Alexa Fluor‐conjugated secondary antibodies that were used for immunofluorescence (IF) included donkey anti‐mouse IgG H&L (Alexa Fluor® 488, ab150105, Abcam), goat anti‐chicken IgG H&L (Alexa Fluor® 647, ab150171, Abcam) and donkey anti‐chicken IgG H&L (Alexa Fluor® 594, 703–585‐155, Jackson). BODIPY 493/503 was purchased from Invitrogen (D3922). DAPI Staining Solution was purchased from Abcam. Protease inhibitors and phosphatase inhibitors were purchased from Bimake, and Triton X‐100 was purchased from Sigma. Donkey serum was purchased from Gibco. Lipopolysaccharide (LPS) (Sigma, L2630) were used to establish the SAE model, ACSF (Leagene, CZ0540) were injected into the lateral ventricle in the control group. The detailed viral sequence is HILPDA mouse, NM 023516.5, shRNA: GCCTCCTAAGGGCCTGCCAGA. The rAAV‐GFAP‐mCherry‐5′miR‐30a‐shRNA(HILPDA)‐3′‐miR30a‐WPREs, AAV2/5 type (shHILPDA), and the rAAV‐GFAP‐mCherry‐5′miR‐30a‐shRNA(scramble)‐3′‐miR30a‐WPREs, AAV2/5 type (control AAV), were used in our experiments and were purchased from Brain VTA Co., Ltd. (Wuhan).

### 2.3 Establishment of the SAE model

In the LPS group, 2 μL of purified LPS solution dissolved by ACSF to 2 mg/mL was injected into the lateral ventricle of mice with a stereotaxic device.^19^, ^20^ And in the ACSF group, equal volume of ACSF was injected into the lateral ventricle. The coordinates were as follows: −2.0 mm dorsoventral (DV), 1.0 mm mediolateral (ML), and − 0.5 mm anteroposterior (AP) to Bregma.

### 2.4 Virus injection

The shHILPDA virus and control AAV were stereotaxically injected into the CA1 region of the hippocampus. First, the mice were anesthetized with 2% pentobarbital sodium (20 mg/kg, i.p.) and fixed on a stereotaxic device. The mouse head was disinfected with iodophor, the skin of the mouse head was cut, and the surface of the skull was cleaned with hydrogen peroxide. Bregma was used as the origin point to flatten the mouse brain. We then injected 300 nL of virus into the CA3 region of the hippocampus (AP = −1.9 mm, ML = ±1.3 mm, DV = −1.75 mm).

### 2.5 Behavioral tests

#### 2.5.1 Open field test

In our experiment, the open field test was used to evaluate the motor abilities of the mice. At the beginning of the experiment, the mice were acclimatized to conditions of 10 lux of light for 2 h and then placed in the same position in a PVC box (40 cm × 40 cm × 40 cm). The activities of the mice were recorded for 5 min with a video behavior analysis system. The locomotor function of the mice was determined by calculating the total distance that the mice traveled. After each test, the PVC box was cleaned with 75% alcohol, and a clean and odorless PVC box was used in the next test.

#### 2.5.2 Contextual fear conditioning test

The contextual fear conditioning test is a behavioral paradigm that is used to reflect learning and memory.^21^ The experiment was divided into the habituation stage, the training stage, and the retrieval stage. All mice were placed in the behavioral test room and allowed to acclimate for 2 h before the experiment. In the habituation stage, the mice were placed in a fear box, and their basic motor ability was recorded for 10 min. In the training stage, after having spent 2 min in the fear box, the mice were administered an electric shock to the foot (0.6 mA current, lasting 2 s) for a total of five times with 1 min intervals between shocks. In the retrieval stage, the mice were placed in the same fear box, and the activities of the mice were recorded for 10 min. The proportion of time spent freezing relative to the total time was calculated over the first 5 min; this value represented the freezing time ratio.

#### 2.5.3 Y‐Maze test

The Y‐maze test is used to stimulate spatial working memory and exploratory activity.^22^ The apparatus consisted of a y‐shaped acrylic maze with three identical arms (34 × 8 × 14 cm). All mice were placed in the behavioral room and allowed to acclimate for 2 h before the experiment. First, the novel arm was blocked with a baffle; the mice were placed in the starting arm and allowed to explore the starting arm and the old arm for 10 min. Then, the mice were removed and placed back in their cages, where they remained for 1 h before the memory retrieval test was performed. During the memory retrieval test, the baffle that was blocking the novel arm was removed, and the mice were allowed to freely explore all three arms. Finally, the time spent in the novel arm and the number of entries into the novel arm were calculated.

### 2.6 Lipid metabolomics

#### 2.6.1 Test methods

Hippocampal tissues (10 mg) were placed in 2 mL centrifuge tubes, and 750 μL of a chloroform: methanol solution (2:1; precooled at −20°C) and two steel balls (the insufficient sample size was reduced to an equal scale) were added. The lipids were extracted using a chloroform: methanol solution. Finally, the samples were dissolved in 200 μL isopropanol, and the supernatants were filtered through a 0.22 μm membrane to obtain the prepared samples that were used for LC–MSMS. LC–MS analysis was performed using a Thermo Ultimate 3000 and Thermo Q Exactive from Gene Denovo Biotechnology Co. (Guangzhou, China).

#### 2.6.2 Analytical methods

Raw data were obtained from LipidSearch software (v4.0; Thermo Scientific; *.raw format). Each lipid was annotated to obtain a data matrix of the mass‐to‐charge ratio (m/z), retention time (rt) and peak intensity. Then, Lipid Search software (v4.0) was used to perform peak identification, peak filtration, and peak alignment for each lipid. The main parameters were as follows: R.T. tolerance = 0.25 and m score threshold = 2. All the peak intensities were batch normalized to the total spectral intensity to compare data of different orders of magnitude. We used partial least squares discriminant ana‐ lysis (PLS‐DA) and orthogonal partial least squares discriminant analysis (OPLS‐DA) to evaluate the difference in metabolic profiles. The significant metabolites with variable important in projection (VIP) ≥ l, and *p* value (*T* test) <0.05.

#### 2.6.3 Western blotting

Hippocampal samples (g) were homogenized in RIPA lysis buffer (μL) containing protease (μL) and phosphatase (μL) inhibitors at a ratio of 1:10,000:100:100. The homogenates were incubated on ice for 20 min and centrifuged at 12,000 rpm for 10 min, and the supernatants were extracted and denatured with 4× loading buffer at 100°C for 10 min. Subsequently, 7 μL of each sample was separated by SDS–PAGE and transferred to a polyvinylidene fluoride (PVDF) membrane. After running at a voltage of 90 V for 60 min, the membranes were blocked with 5% skim milk for 1 h. Then, the membranes were incubated with the primary antibodies overnight at 4°C, washed three times with TBST for 5 min each and incubated with the secondary antibodies at room temperature for 2 h. After washing with TBST, the membranes were developed with chemical reagents, and images were captured with a Bio‐Rad gel imaging system.

#### 2.6.4 IF staining

Frozen sections (12 μm thick) of mouse hippocampal tissues were generated. The brain sections were washed 3 times with phosphate‐buffered saline (PBS) for 5 min each. Then, the sections were blocked with donkey serum and 0.05% Triton X‐100 at room temperature for 2 h. The sections were incubated with primary antibodies at 4°C for 36 h. After thorough washing, the sections were incubated with a secondary antibody in the dark for 2 h and then stained with DAPI for 10 min.

#### 2.6.5 Transmission electron microscopy (TEM)

Mice were anesthetized with 2% pentobarbital sodium (20 mg/kg) and transcardially perfused with 10 mL of saline, followed by 50 mL of ice‐cold mixture of 2% paraformaldehyde and 2% glutaraldehyde in 0.1 M phosphate buffer (PB) for 1 h. The brains were removed and postfixed by immersion in the same fixative for 4 h at 4°C. Serial coronal sections (50 μm thick) were prepared with a vibratome (VS1000s, Leica). The sections were postfixed in 1% osmium tetroxide in 0.1 M PB for 2 h. Then, the sections were dehydrated with graded ethanol, incubated with propylene oxide, and finally embedded in Epon 812 between plastic sheets. After polymerization, flat‐embedded sections were examined under a light microscope. The hippocampal CA1 region was selected, trimmed under a stereomicroscope, and then glued onto blank resin stubs. Serial ultrathin sections were cut with a diamond knife (Diatome, Hatfield, PA) and an ultramicrotome (Leica EM UC6, Wetzlar, Germany) and mounted on Formvar‐coated mesh grids (6–8 sections/grid). The sections were then counterstained with uranyl acetate and lead citrate for electron microscopic examination. The ultrathin sections were examined under a JEM‐1230 electron microscope (JEOL LTD, Tokyo, Japan) equipped with a CCD camera and its application software (832 SC1000, Gatan, Warrendale, PA).

### 2.7 Statistical analysis

We assessed the normality and variance homogeneity by GraphPad Prism 8.0.2 software, the Shapiro–Wilk test showed that *p* > 0.05 was consistent with normal distribution. Bonferroni or Dunn's post hoc analysis was used to determine where those differences occurred. We compared every two groups with a two‐tailed Student's *t* test. All data are presented as the mean ± SEM. The criterion for statistical significance was *p* < 0.05.

## 3 RESULTS

### 3.1 Seventy‐two hours after LPS was injected into the lateral ventricle, the mice showed severe cognitive dysfunction

**FIGURE 1:**
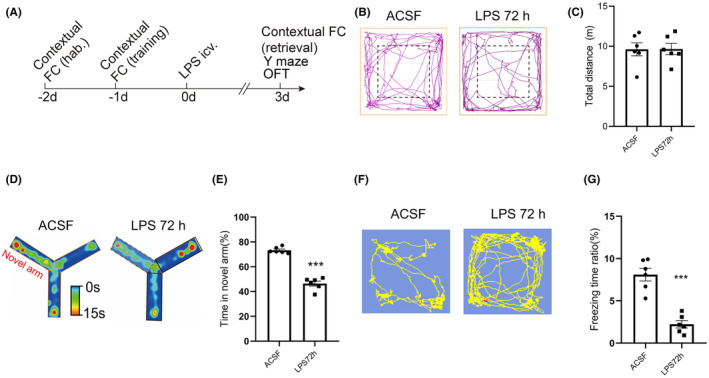
Cognitive dysfunction occurs in mice with SAE. (A) Summary of the experimental procedures used for modeling and the behavioral tests. (B) Representative tracks of mice in the ACSF group and LPS group in the open field test. (C) Total distance traveled by mice in the ACSF and LPS groups (ACSF: *n* = 6; LPS: *n* = 6, *t* = 0.03, *p* = 0.97). (D) Representative heatmap of mice in the ACSF and LPS groups in the Y‐maze test. (E) Time spent in the novel arm in the Y‐maze test (ACSF: *n* = 6; LPS: *n* = 6, *t* = 11.90, *p* < 0.0001). (F) Representative tracks of mice in the ACSF and LPS groups in the contextual fear conditioning test. (G) Freezing time ratio (%) of the ACSF and LPS groups (ACSF: *n* = 6; LPS: *n* = 6, *t* = 6.81, *p* < 0.0001). The Student's *t* test was used for statistical analysis. ***p < 0.001. All data are shown as the mean ± SEM.

On the third day after the model was established, a behavioral test was performed to determine whether SAE mice developed cognitive dysfunction (Figure 1A). The locomotor function of the mice was evaluated with the open field test (Figure 1B), and the results showed that the total distance traveled was not different between the ACSF and LPS groups (ACSF 9.62 ± 0.81 vs. LPS 9.68 ± 0.69, Figure 1C). The Y‐maze test showed that the time spent in the novel arm (% of total time) was significantly shorter in the LPS group than in the ACSF group (ACSF 73.24 ± 1.01 vs. LPS 46.37 ± 2.02, Figure 1D,E). The contextual fear conditioning test showed that the freezing time ratio was significantly decreased in the LPS group (ACSF 8.09 ± 0.74 vs. LPS 2.22 ± 0.44, Figure 1F,G). These results indicated that 72 h after injecting LPS into the lateral ventricle, the mice developed cognitive dysfunction.

### 3.2 Lipid metabolism homeostasis was widely disrupted in the hippocampus of SAE mice

**FIGURE 2:**
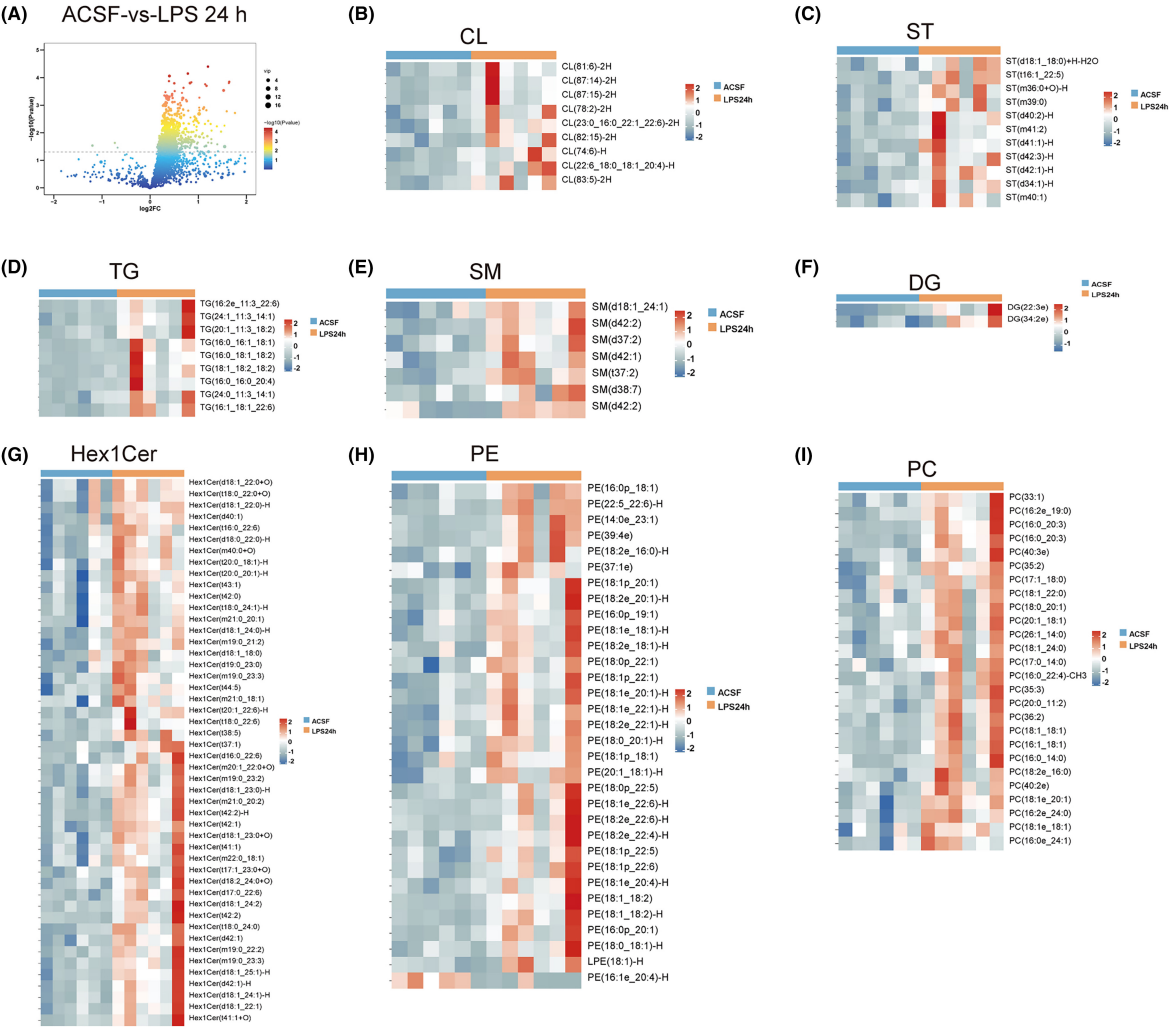
Lipid metabolism in hippocampal tissue at 24 h after LPS injection into the lateral ventricle. (A) Volcano plot of the ACSF and LPS 24 h groups. (B) The heatmap of cardiolipin (CL). (C) Heatmap of sulfatide (ST). (D) Heatmap of triglycerides (TGs). (E) Heatmap of sphingomyelin (SM). (F) Heatmap of diglycerol (DG). (G) Heatmap of hexosylceramide (Hex1Cer). (H) Heatmap of phosphatidylethanolamine (PE). (I) Heatmap of phosphatidylcholine (PC).

**FIGURE 3:**
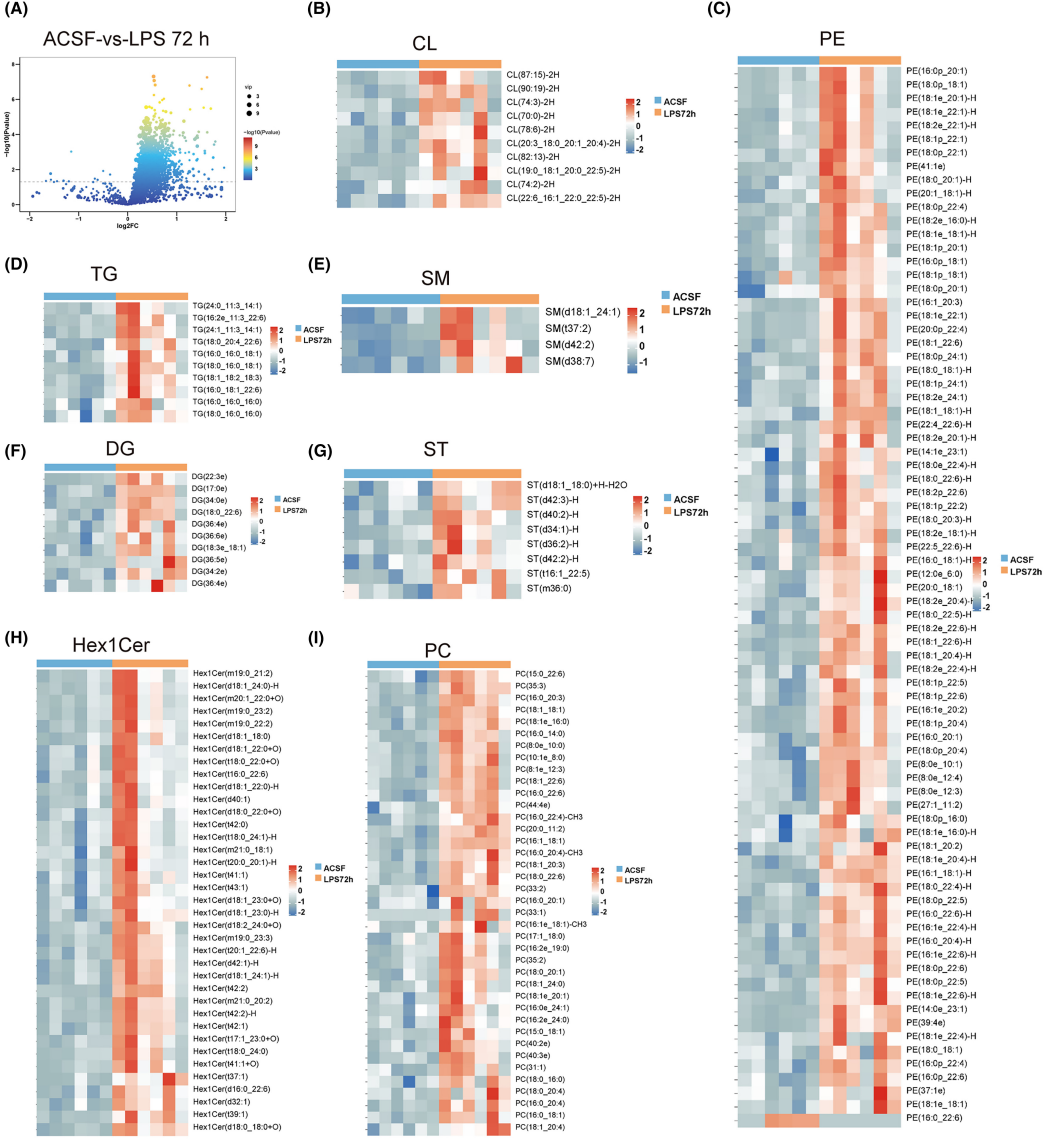
Lipid metabolism in hippocampal tissue at 72 h after LPS injection into the lateral ventricle. (A) Volcano plot of the ACSF and LPS 72 h groups. (B) The heatmap of cardiolipin (CL). (C) Heatmap of phosphatidylethanolamine (PE). (D) Heatmap of triglycerides (TGs). (E) Heatmap of sphingomyelin (SM). (F) Heatmap of diglycerol (DG). (G) Heatmap of sulfatide (ST). (H) Heatmap of hexosylceramide (Hex1Cer). (I) Heatmap of phosphatidylcholine (PC).

**FIGURE 4:**
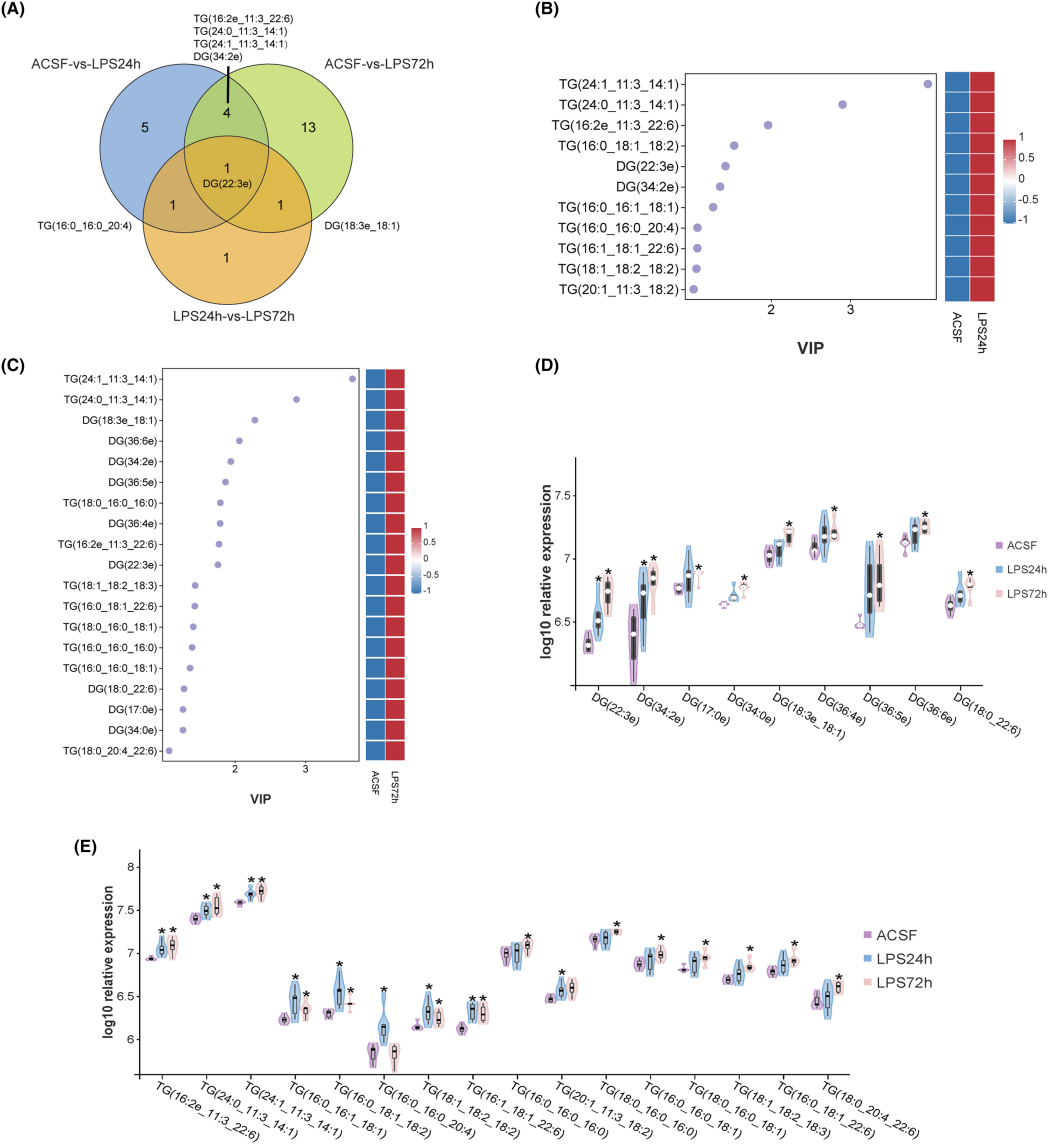
The neutral lipids in the TG and DG were different at different time points after LPS injection. (A) Venn diagrams of neutral lipids among the three groups. (B) Variable importance in projection (VIP) diagram of OPLS‐DA for neutral lipids between ACSF‐vs.‐LPS24h. (C) Variable importance in projection (VIP) diagram of OPLS‐DA for neutral lipids between ACSF‐vs.‐LPS72h. (D) Differences in DG among the three groups. (E) Differences in TG among the three groups. *P < 0.05.

Studies have shown that lipid metabolism disorders are closely related to neurodegenerative diseases.^23^ To explore whether lipid metabolism was altered in SAE mice, lipid metabolomics was used to analyze lipid metabolism in the hippocampus. Our results showed that lipid metabolism was widely altered (Figures 2A and 3A). The expression of phospholipids, such as cardiolipin (CL), phosphatidylcholine (PC) and phosphatidylethanolamine (PE) increased at 24 h. This increase was most significant at 72 h (Figures 2B,H,I and 3B,C,I). The expression of neutral lipids, including diglycerol (DG) and triglyceride (TG), did not change significantly at 24 h but increased significantly at 72 h (Figures 2D,F and 3D,F). The expression of sphingolipids such as hexosylceramide (Hex1cer), sphingomyelin (SM) and sulfatide (ST) was significantly increased at 24 h and 72 h (Figures 2C,E,G and 3E,G,H). To further clarify the changes in neutral lipids metabolism, we conducted a more in‐depth analysis of these two periods. The Venn diagram shows the relationship between three groups of neutral lipids (Figure 4A). At the same time, VIP (variable importance in projection) values was utilized for the presentation of DG and TG subtypes' variables, which are crucial and contribute significantly to distinguishing between the groups (Figure 4B,C). A comprehensive analysis of the three groups revealed differences in the subtypes and quantities of neutral lipids TG and DG at different time points during LPS modeling (Figure 4D,E). These results suggested that the homeostasis of hippocampal lipid metabolism was widely disrupted 72 h after the onset of SAE.

### 3.3 LDs accumulated in astrocytes of the hippocampus

**FIGURE 5:**
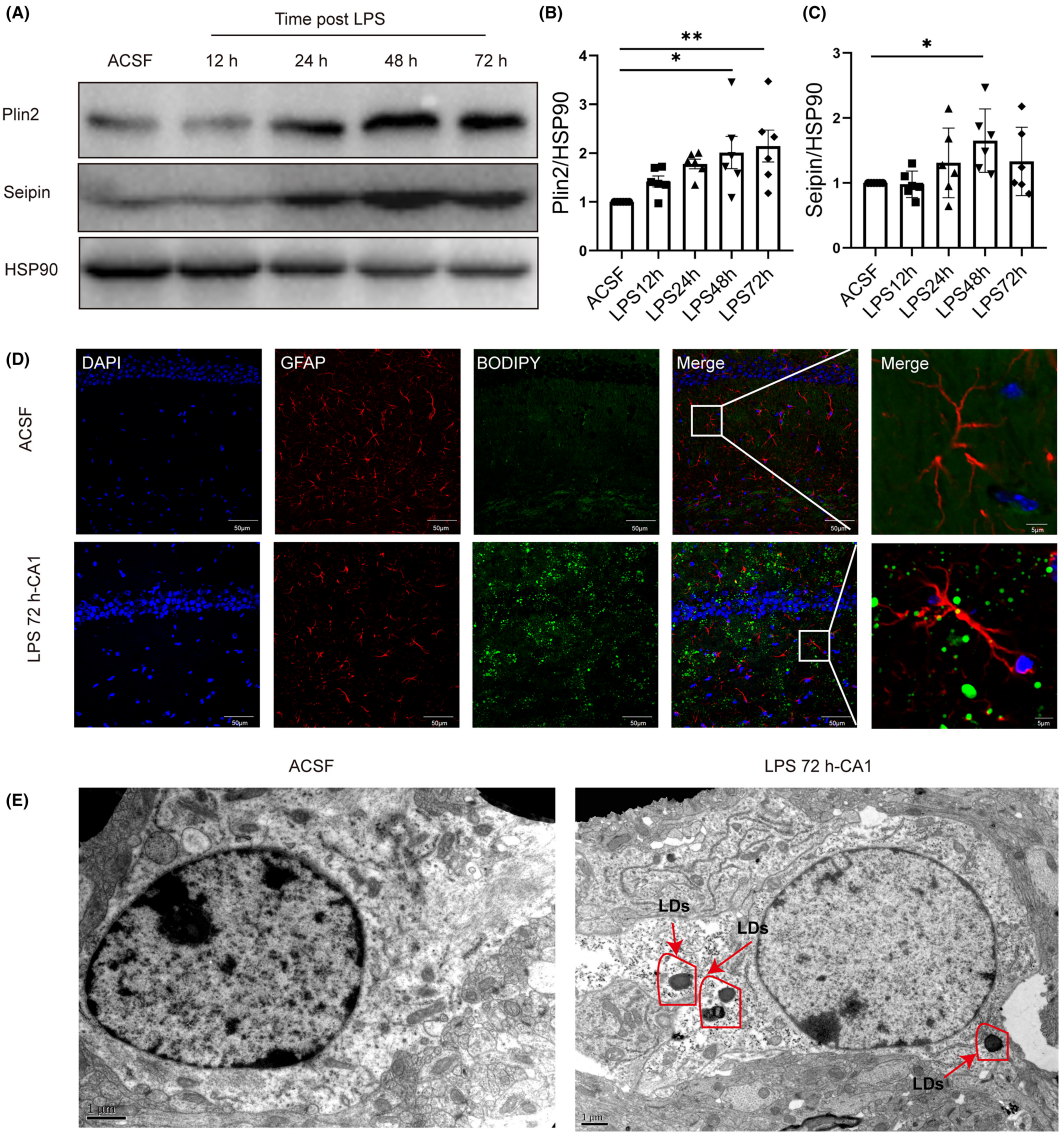
LDs accumulation within astrocytes of the hippocampus. (A) Representative Westering blot of Plin2 and Seipin. Quantification of (B) Plin2 (*n* = 6, *F* = 4.52, *p = 0.01, **p = 0.004, one‐way ANOVA was used for statistical analysis.) and (C) Seipin (*n* = 6, *F* = 2.74, *p = 0.04, one‐way ANOVA was used for statistical analysis.) levels measured as the ratio of the target band density to the HSP90 band density. (D) IF staining of BODIPY 72 h after injection of LPS into the lateral ventricle (bar = 50 μm or 5 μm). (E) Representative TEM images of tissues from mice treated with ACSF and LPS (bar = 1.0 μm); the red arrows represent lipid droplets. *p < 0.05, **p < 0.01, compared with the ACSF group. All data are shown as the mean ± SEM.

LDs are organelles of lipid storage, while lipid metabolism disorders may affect LDs production.^24^ To determine whether the LDs levels were increased, the expression of the LDs related proteins Plin2 and seipin was measured by Western blotting. The results revealed that the expression of Plin2 was significantly increased at 48 h and 72 h compared with that in the ACSF group (ACSF 1 vs. LPS 48 h 2.01 ± 0.33 vs. LPS 72 h 2.15 ± 0.33, Figure 5A,B). The expression of seipin was significantly increased at 48 h (ACSF 1 vs. LPS 48 h 1.65 ± 0.20, Figure 5A,C). These results indicated that in addition to DG and TG changes, LDs formation was also significantly increased in the hippocampus of SAE mice induced by LPS.

As previously reported, the CA1 hippocampal region is mainly involved in the regulation of cognition.^25^ In this study, we found that in the CA1 region of the hippocampus of mice, LDs significantly accumulated in astrocytes (Figure 5D). TEM further confirmed that LDs accumulated in astrocytes of the hippocampus 72 h after LPS was injected into the lateral ventricle (Figure 5E).

### 3.4 HILPDA is the key mediator of LDs formation in hippocampal astrocytes

**FIGURE 6:**
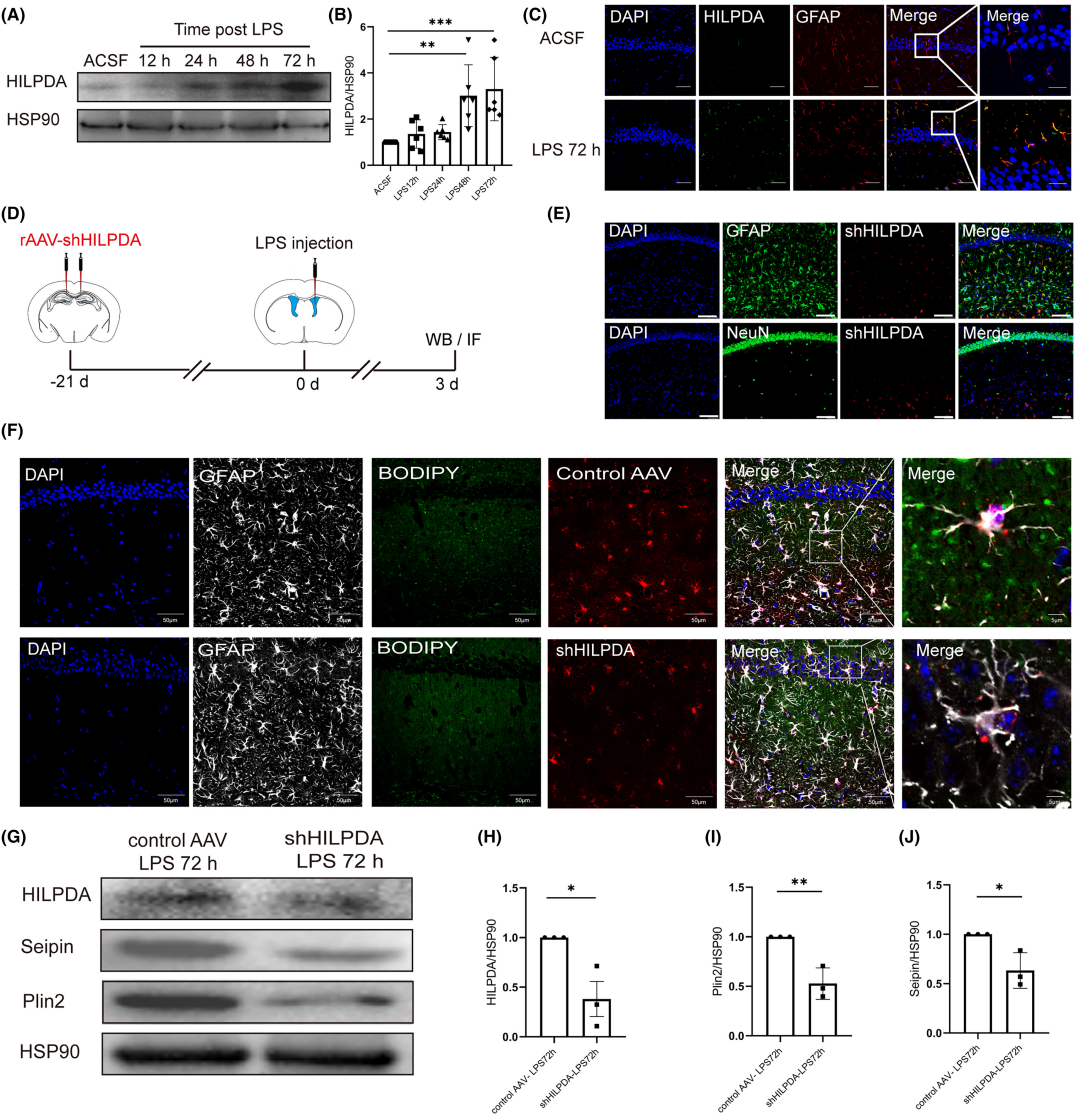
HILPDA is a key protein that mediates LDs formation in hippocampal astrocytes of SAE mice. (A) Representative Western blot of HILPDA. (B) Quantification of HILPDA levels measured as the ratio of the target band density to the HSP90 band density (*n* = 6, *F* = 8.03, **p = 0.003, ***p = 0.0007, one‐way ANOVA was used for statistical analysis.). **p < 0.01, ***p < 0.001, compared with the ACSF group. (C) IF staining of HILPDA 72 h after injection of LPS into the lateral ventricle (bar = 50 μm or 20 μm). (D) Summary of the experimental procedures used for shHILPDA injection. (E) To verify the efficiency of shHILPDA expression (bar = 50 μm). (F) IF staining of BODIPY 72 h after injection of LPS into the lateral ventricle (bar = 50 μm or 5 μm). (G) Representative Western blot of HILPDA, Plin2, and Seipin. (H) Quantification of HILPDA levels measured as the ratio of the target band density to the HSP90 band density (*n* = 3, *t* = 3.49, *p* = 0.025, The Student's *t* test was used for statistical analysis.). (I) Quantification of plin2 levels measured as the ratio of the target band density to the HSP90 band density (*n* = 3, *t* = 5.13, *p* = 0.0068, The Student's *t* test was used for statistical analysis.). (J) Quantification of Seipin levels measured as the ratio of the target band density to the HSP90 band density (*n* = 3, *t* = 3.54, *p* = 0.0241, The Student's *t* test was used for statistical analysis.). *p < 0.05, **p < 0.01. All data are shown as the mean ± SEM.

HILPDA, which is a lipid droplet‐associated protein, governs the formation and degradation of LDs.^26^ Therefore, it is crucial to explore whether HILPDA mediates LDs formation in astrocytes. As demonstrated in Figure 6, the expression of HILPDA in the hippocampus was measured at 12 h, 24 h, 48 h, and 72 h after LPS injection. The Western blotting results showed that the expression of HILPDA was significantly increased at 48 h and 72 h (ACSF 1 vs. LPS 48 h 3.02 ± 0.55 vs. LPS 72 h 3.30 ± 0.56, Figure 6A,B).

Moreover, the IF results also confirmed that the expression of HILPDA was significantly increased 72 h in hippocampal astrocytes after LPS was injected (Figure 6C). To determine the key role of HILPDA in LDs formation in the astrocytes of SAE mice, the shHILPDA virus, which specifically downregulates astrocytic HILPDA, was injected into the CA1 region of the hippocampus (Figure 6D). The efficiency of virus delivery was confirmed by Western blotting (Figure 6G,H). By using the neuronal marker NeuN and the astrocyte marker GFAP, we confirmed that HILPDA was specifically downregulated in astrocytes (Figure 6E,F). The Western blotting results showed that the expression of plin2 and seipin was significantly decreased after the specific downregulation of HILPDA, suggesting a decrease in LDs formation (Figure 6G,I,J).

### 3.5 Inhibition of astrocytic LDs formation ameliorated cognitive dysfunction in SAE mice

**FIGURE 7:**
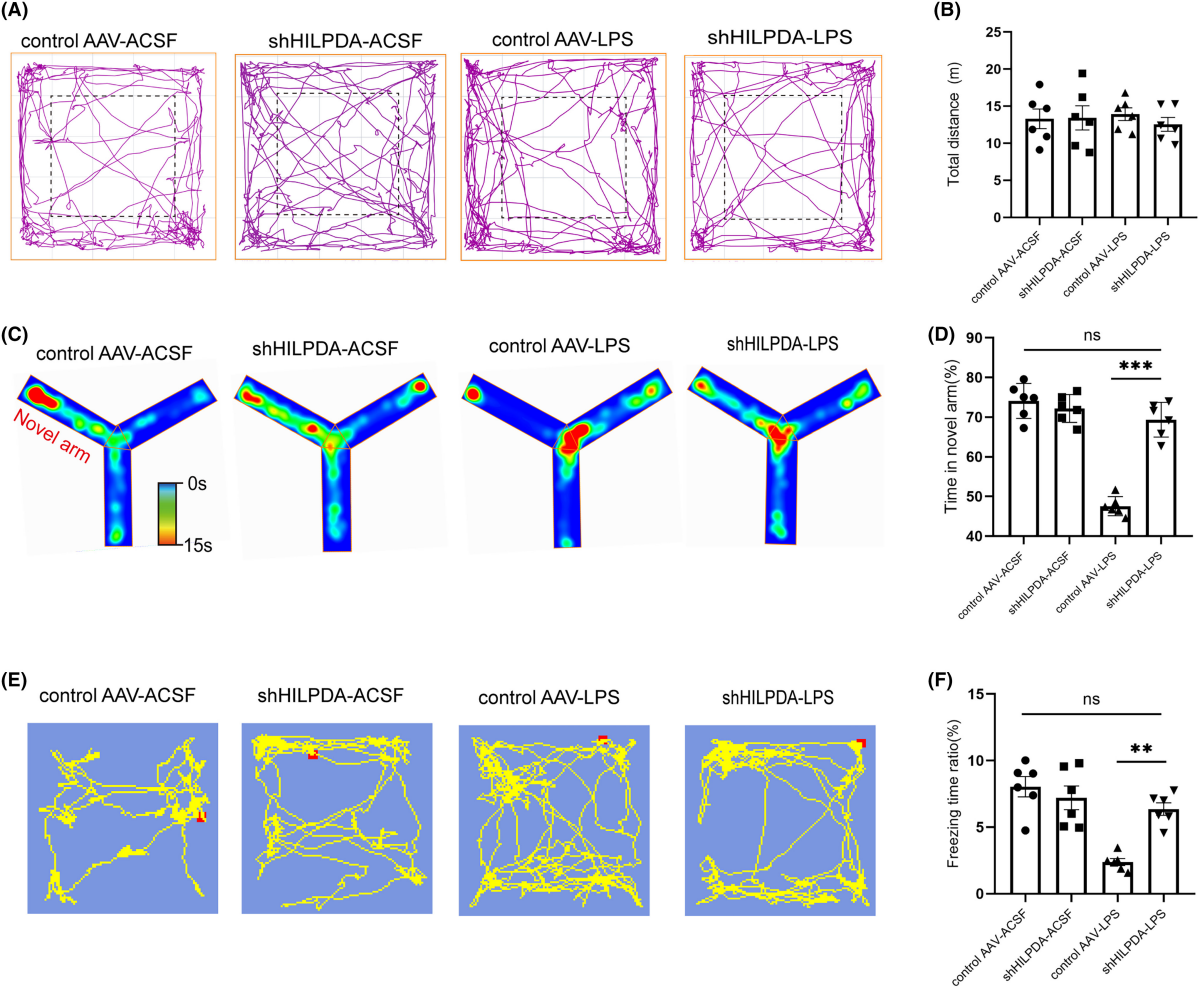
Inhibition of LDs formation can ameliorate cognitive dysfunction in SAE mice. (A) Representative tracks of mice in the different groups in the open field test. (B) Total distance traveled by mice in the different groups (*n* = 6, *F*(1, 20) = 0.37). (C) Representative heatmap of mice in the different groups in the Y‐maze test. (D) The time spent in the novel arm by mice in the different groups in the Y‐maze test (*n* = 6, *F*(1, 20) = 59.78, ***p < 0.001). (E) Representative tracks of mice in the different groups in the contextual fear conditioning test. (F) Freezing time ratios (%) of the different groups (*n* = 6, *F*(1, 20) = 14.09, **p = 0.0017). **p < 0.01, ***p < 0.001. Two‐way ANOVA with Sidak's multiple comparisons test was used for statistical analysis. All data are shown as the mean ± SEM.

To confirm whether LDs accumulation in astrocytes leads to cognitive dysfunction in SAE mice, the open field test was performed to exclude the possibility of locomotor dysfunction. The results showed that there was no difference in the total distance traveled among all the groups (Figure 7A,B). The Y‐maze test showed that compared with the LPS group, the time spent in the novel arm (% of total time) was significantly longer after the injection of shHILPDA (control AAV‐LPS 47.55 ± 0.99 vs. shHILPDA‐LPS 69.37 ± 1.79, Figure 7C,D), suggesting that inhibiting astrocytic LDs accumulation could alleviate spatial learning and memory impairment. The contextual fear conditioning test revealed that compared with the LPS group, the freezing time ratio was significantly higher after the injection of shHILPDA (control AAV‐LPS 2.38 ± 0.26 vs. shHILPDA‐LPS 6.36 ± 0.47, Figure 7E,F), suggesting that reducing LDs accumulation could alleviate contextual association memory impairment in SAE mice. These results indicate that the accumulation of LDs in astrocytes is one of the key pathological mechanisms that leads to cognitive dysfunction in SAE mice.

### 3.6 Inhibition of astrocytic LDs accumulation attenuated cognitive dysfunction in SAE mice by inhibiting the conversion of astrocytes to the A1 phenotype and reducing neuronal membrane potential excitability

**FIGURE 8:**
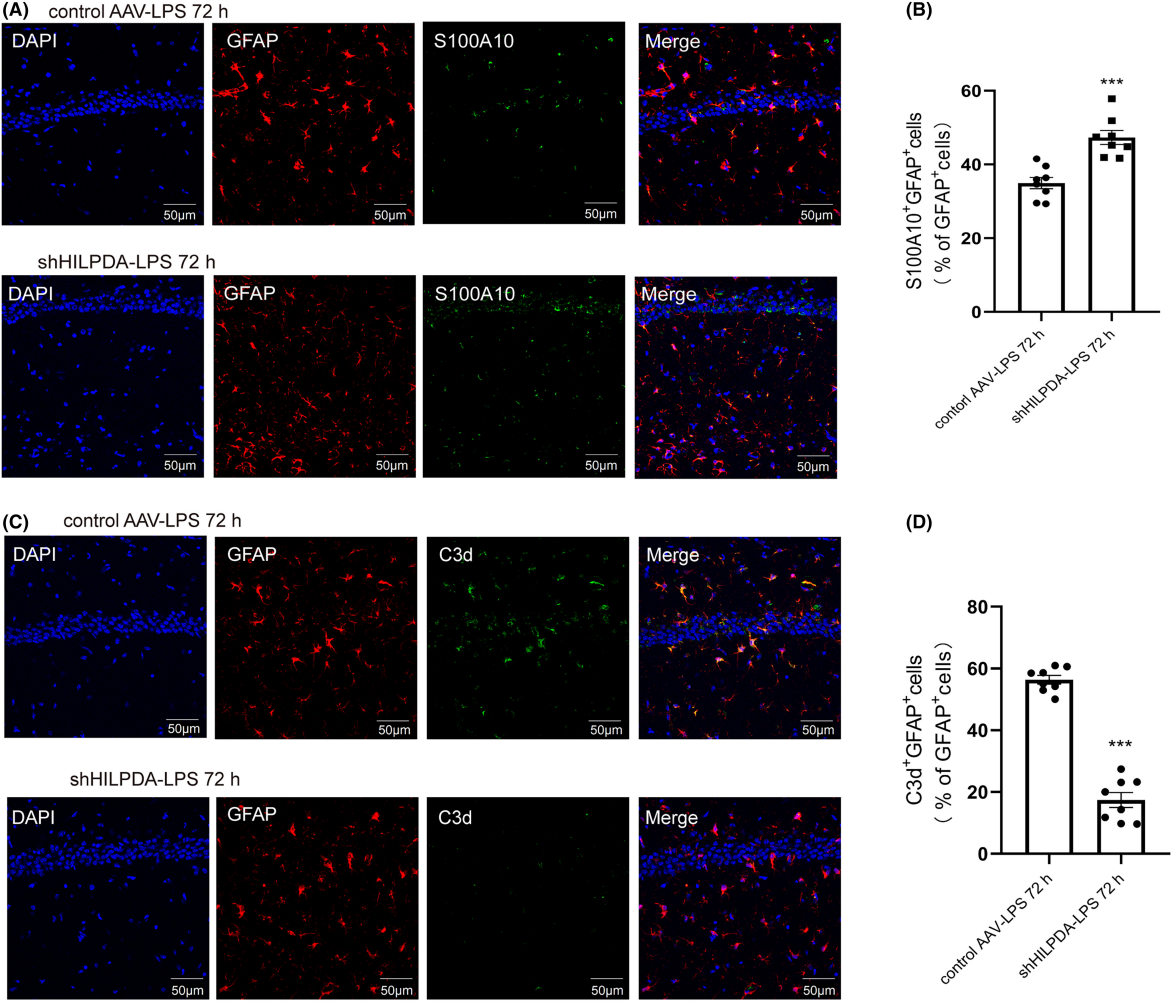
Inhibition of astrocytic LDs accumulation can reduce the conversion of astrocytes to the A1 phenotype. (A) IF staining of GFAP and S100A10 72 h after injection of LPS into the lateral ventricle (bar = 50 μm). (B) Percentage of S100A10^+^ GFAP^+^ cells relative to GFAP^+^ cells in the left panel (*n* = 8, *t* = 5.04, ***p = 0.0002). (C) IF staining of GFAP and C3d 72 h after injection of LPS into the lateral ventricle (bar = 50 μm). (D) Percentage of C3d ^+^ GFAP^+^ cells relative to GFAP^+^ cells in the left panel (*n* = 8, *t* = 13.87, ***p < 0.001). ***p < 0.001. The Student's *t* test was used for statistical analysis. All data are shown as the mean ± SEM.

Since astrocytes have A1 and A2 phenotypes after activation, we measured the transformative status of astrocytes after astrocytic LDs accumulation was inhibited. Our IF results showed that in the shHILPDA‐LPS group, the expression of S100A10^+^ GFAP^+^ was increased (control AAV‐LPS 34.95 ± 1.55 vs. shHILPDA‐LPS 47.31 ± 1.90, Figure 8A,B), while C3D^+^ GFAP^+^ was significantly decreased (control AAV‐LPS 56.36 ± 1.39 vs. shHILPDA‐LPS 17.40 ± 2.44, Figure 8C,D).

**FIGURE 9:**
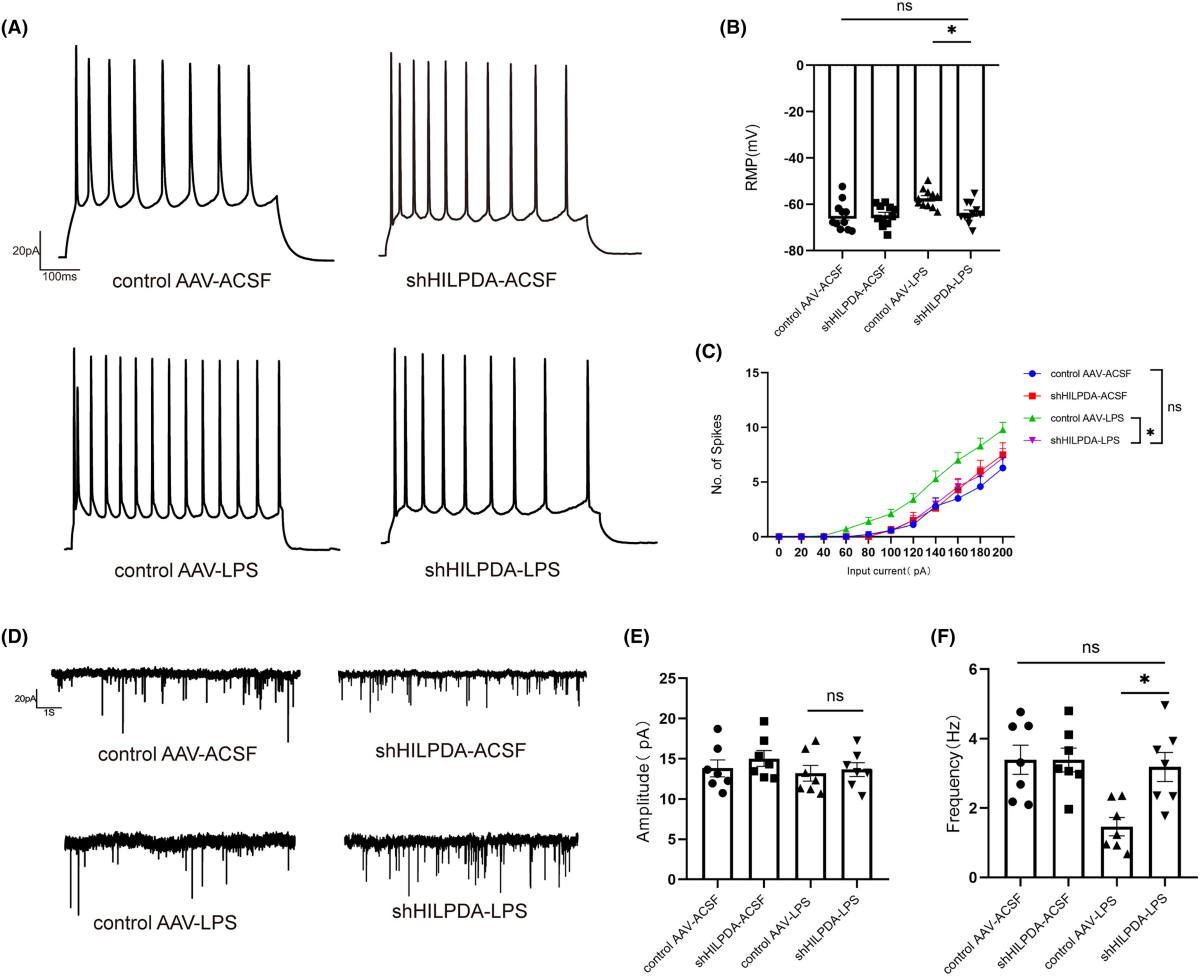
Inhibition of astrocytic LDs accumulation decreases neuronal membrane potential excitability. (A) Firing activity in response to +100 pA depolarizing current intracellular injection of control AAV‐ACSF, shILPDA‐ACSF, control AAV‐LPS and shHILPDA‐LPS. (B) Mean RMP of CA1 pyramidal neurons (*n* = 11, *F*(1, 40) = 4.86, *p = 0.025, Two‐way ANOVA with Sidak's multiple comparisons test was used for statistical analysis.). (C) Number of spikes in response to different depolarizing currents in the four groups of CA1 pyramidal neurons (*n* = 11, *F*(30, 360) = 2.28, *p = 0.0118, Two‐way repeated ANOVA with Bonferroni's post hoc test was used for statistical analysis). (D) Samples of sEPSCs recorded in CA1 pyramidal neurons. (E, F) Mean amplitude (left, *n* = 7, *F*(1, 24) = 0.156, Two‐way ANOVA with Sidak's multiple comparisons test was used for statistical analysis.) and frequency (right, *n* = 7, *F*(1, 24) = 5.54, *p = 0.017, Two‐way ANOVA with Sidak's multiple comparisons test was used for statistical analysis.) of sEPSCs of CA1 pyramidal neurons. *p < 0.05. All data are shown as the mean ± SEM.

To further confirm these results, we employed patch‐clamp recording of vertebral neurons in hippocampal CA1 and revealed that the excitability of neurons was elevated after LPS. Specifically, the known regulation of astrocytic HIPDA reversed this phenomenon (RMP control AAV‐LPS ‐57.45 ± 1.20 vs. shHILPDA‐LPS ‐63.78 ± 1.37, Figure 9A–C). In addition, the EPSC frequency of hippocampal vertebral neurons decreased after LPS, indicating that the synaptic connections of hippocampal vertebral neurons could be reduced after LPS, and the same phenomenon can also be reversed after downregulating astrocytic HIPDA (Frequency control AAV‐LPS 1.47 ± 0.26 vs. shHILPDA‐LPS 3.19 ± 0.42, Figure 9D–F). The above results suggest that inhibition of astrocytic LDs accumulation ameliorates cognitive dysfunction in SAE mice by inhibiting the conversion of astrocytes to the A1 phenotype and neuronal membrane potential excitability.

## 4 DISCUSSION

In the current study, we explored the role of astrocytic LDs in cognitive dysfunction in SAE mice, as well as the underlying mechanism. We confirmed that LDs accumulate in hippocampal astrocytes. We proved that specific downregulation of astrocytic HILPDA reduced the accumulation of LDs. In addition, inhibition the transformation of astrocytes to the A1 phenotype inhibited the release of presynaptic excitatory transmitters and ameliorated contextual association memory and spatial learning memory in SAE mice.

SAE is one of the most serious complications of sepsis.^27^ The mortality rate reaches 70%, while 60% of surviving patients have cognitive impairment.^28^ Clinically, Semmler A. et al. have also demonstrated that sepsis survivors develop cognitive dysfunction and showed a significant decrease in hippocampal volume in SAE survivors.^5^ In accordance with this clinical evidence, we established an SAE mouse model by injecting LPS into the lateral ventricle of mice and proved that contextual association memory and spatial learning memory ability were impaired.

In our experiment, the levels of TG and DG in the hippocampus of SAE mice were found to be significantly increased after the injection of LPS. These results suggested that lipid metabolism was altered in the hippocampal tissues of SAE mice. Chen et al. demonstrated that the accumulation of lipids in astrocytes leads to reactive astrocyte development, resulting in the increased expression of APOE, and the knockdown of APOE suppresses seizure activity.^29^ Accordingly, Liu CC et al. suggested that APOE expression in the liver impairs synaptic plasticity and cognition by inhibiting cerebrovascular function in a mouse model of Alzheimer's disease.^30^ Therefore, cognitive impairment in SAE mice might be related to abnormal lipid metabolism.

LDs are storage organelles involved in lipid and energy homeostasis, and lipid metabolism disorders can affect LDs production.^12^ In this study, we examined the LDs‐associated protein Plin2 and found that its expression was significantly increased 72 h after the onset of LPS injection. BODIPY, which is a marker of LDs, was confirmed to accumulate in astrocytes 72 h after establishment. ApoE is the most abundant lipoprotein in the brain, and it has been suggested that glial cell‐derived APOE4 affects learning and memory by regulating neuronal lipid metabolism.^31^ Furthermore, Li Q et al. confirmation that microglia lipid droplet accumulation is associated with diabetes‐related cognitive dysfunction.^32^ These evidence suggests that lipid metabolism plays a key role in learning and memory deficits in neurodegenerative diseases.

In the current study, we used chemical and genetic methods to inhibit LDs accumulation in hippocampal astrocytes. Our results showed that inhibiting the formation of LDs could ameliorate learning and memory impairment in SAE mice. Astrocytes and neurons have many interactions in the brain.^33^ Paumier A et al. confirmed that the intraperitoneal administration of a specific inhibitor of TRPV1 channels can lead to hippocampal astrocyte overactivation, prevent neuronal dysfunction and maintain structural synaptic integrity to ameliorate spatial working memory in Alzheimer's disease.^34^ In addition, the A1 astrocytes secrete a variety of inflammatory cytokines, resulting in neuronal apoptosis.^35^ Our results showed that inhibition of LDs production inhibited the conversion of astrocytes to the A1 phenotype. Furthermore, previous studies have suggested that after neurons are overexcited, astrocyte lipid droplets increase.^10^ In accordance with this phenomenon, our patch‐clamp recording of vertebral neurons in hippocampal CA1 revealed that the excitability of neurons was elevated after LPS, while downregulation of HIPDA in astrocytes reversed this phenomenon.

There are several limitations in our study. Firstly, the switch between A1 and A2 phenotypes of astrocyte has not been explored in depth, but some research reported that LDs induce endoplasmic reticulum stress and then induce the conversion of the A1 phenotype and the A2 phenotype. Secondly, the patients of SAE are predominant elderly population, while young mice were used in this experiment. Thus, the mechanism of SAE demonstrated in the current study need to be further verified in elderly mice. Finally, we established SAE mice model by LPS intracerebroventricular injection to mimic the acute neuroinflammation in response of sepsis. We did not perform a sepsis model that established in previous published articles such as cecal ligation and puncture (CLP)^36^, ^37^ or LPS intraperitoneal injection,^38^ which have been proven to lead to systemic lipid metabolism disorders.^39^ And this will have implications for our exploration of lipid alterations in the hippocampus in SAE. The establishment of a more reasonable pathophysiological model of sepsis has recently been proposed as the primary scientific problem to be solved by the Guideline Working Group of the Surviving Sepsis Campaign.^40^ Through long‐term exploration, our team found that LPS intracerebroventricular injection is stable and repeatable for behavior tests related to emotion and memory.^2^, ^41^ This finding is consistent with the results of studies conducted by various research groups on mechanisms and therapeutic targets associated with neuroinflammation. The priority of our study was to investigate the relationship between neuroinflammation of SAE and lipid metabolism in the central nervous system (CNS). However, the process of fat mobilization and glycogenolysis caused by the systemic inflammatory model may obscure the lipid changes in the CNS. Therefore, we chose the LPS intracerebroventricular injection that can steadily induce neuroinflammation to further explore the relationship between SAE and lipid metabolism.

The model used in this study only simulates the changes of a single organ in sepsis, and its applicability in patients with clinical SAE still needs to be further studied.

In summary, our current research demonstrate the importance of astrocytic lipid droplets in the pathological process of SAE and reveal HILPDA, which is an attractive therapeutic target for lipid metabolism regulation and cognitive improvement in septic patients.

## AUTHOR CONTRIBUTIONS

Ling Li completed the experiments; Ling Li and Lixia Du wrote the manuscript; Xijing Zhang and Zongping Fang guided the project and revised the article; Guifen Gan, Jin Li, You Wu and Lin Yang participated in the analysis of the data.

## FUNDING INFORMATION

This work was supported by grants from the National Natural Science Foundation of China (82272190, 81871603) and the Key Research and Development Plan of Shaanxi Province of China (2023‐ZDLSF‐04) to Xijing Zhang, and the National Natural Science Foundation of China (82171322, 82371328) and the Natural Science Foundation of Shaanxi Province of China (2022KJXX‐102) to Zongping Fang.

## CONFLICT OF INTEREST STATEMENT

The authors declare that they have no competing interests.

## CONSENT FOR PUBLICATION

Not applicable.

## Data Availability

Data relevant to the current study can be obtained from our corresponding authors.
